# Precision Oncology Strategy in Trastuzumab-Resistant Human Epidermal Growth Factor Receptor 2–Positive Colon Cancer: Case Report of Durable Response to Ado-Trastuzumab Emtansine

**DOI:** 10.1200/PO.16.00055

**Published:** 2017-06-27

**Authors:** Derrick S. Haslem, Hanlee P. Ji, James M. Ford, Lincoln D. Nadauld

**Affiliations:** **Derrick S. Haslem** and **Lincoln D. Nadauld**, Dixie Regional Medical Center, Saint George, UT; **Hanlee P. Ji**, **James M. Ford**, and **Lincoln D. Nadauld**, Stanford University School of Medicine, Stanford, CA.

## INTRODUCTION

Colorectal cancer is the third most common cancer and the fourth leading cause of cancer death worldwide.^1^ Metastatic disease is ultimately identified in the majority of patients with colorectal cancer and, with rare exceptions, is considered incurable. The treatment of metastatic colorectal cancer (mCRC) has historically included the use of fluorouracil in combination with other chemotherapeutic agents, including oxaliplatin or irinotecan. The addition of the anti–vascular endothelial growth factor antibody, bevacizumab, to standard chemotherapy regimens has improved the progression-free and overall survival of patients with mCRC and has signaled a shift toward the addition of biologic and targeted agents in the treatment of advanced cancer.^2,3^ The transition toward molecularly targeted therapies has gained additional momentum with the US Food and Drug Administration approval of the anti–epidermal growth factor receptor agents cetuximab and panitumumab for the treatment of *KRAS* wild-type colorectal cancers.^4,5^ Unfortunately, many patients with mCRC progress through these therapies, or are not candidates to receive them on the basis of the molecular profile of their particular tumor, and are left with few treatment options. This common scenario, in addition to advances in molecular testing, has accelerated the application of precision oncology, which uses molecular profiling of a patient’s tumor to identify potentially actionable mutations to enable targeted treatment.^6^

The precision oncology approach has long shown efficacy in molecularly defined subsets of breast (human epidermal growth factor receptor 2 [HER2]) and lung (epidermal growth factor receptor, anaplastic lymphoma kinase) cancers.^7-9^ HER2 assessments in colon cancer have provided evidence regarding the prevalence of these alterations in mCRC and emerging studies have suggested that patients with mCRC may experience prolonged responses to anti-HER2 treatments, indicating that targeted therapy in HER2-positive colon cancer could be a viable approach.^10-13^ Recent advances in next-generation sequencing and digital polymerase chain reaction (PCR) technologies have increasingly allowed for a more refined molecular characterization of a patient’s tumor, including mCRC, thereby enabling precision oncology applications.^14,15^ Accumulating data regarding the efficacy and outcomes of patients treated with molecularly directed therapies is an important next step to understanding the clinical role of precision oncology.

### CASE REPORT

A 48-year-old patient presented to the emergency department complaining of right flank pain and bloody stools for 1 week. Initial laboratory findings revealed a hemoglobin level of 7.1 g/dL. Contrast-enhanced computed tomography (CT) imaging identified thickening of the sigmoid colon and multiple hepatic and pulmonary nodules. A tissue sample from an image-guided core needle biopsy of a liver nodule revealed adenocarcinoma consistent with metastatic colorectal cancer. Molecular analysis confirmed *KRAS* wild-type disease. The patient was previously healthy and did not have a family history of colorectal cancer or other cancer type to suggest an inherited syndrome such as hereditary nonpolyposis colon cancer (HNPCC). The tumor exhibited normal expression of the mismatch repair genes *MLH1*, *MSH2*, *MSH6*, and *PMS2*.

Shortly after diagnosis, the patient began palliative chemotherapy with capecitabine and oxaliplatin every 3 weeks. Bevacizumab was not given because of the presence of rectal bleeding and a history of deep venous thrombosis. Three months after initiation of treatment, an interval CT scan confirmed stable disease without change in size or number of hepatic and pulmonary metastases. However, follow-up imaging 6 months after treatment initiation identified progressive disease in the liver and lung, as well as a new adrenal gland metastasis.

The patient’s treatment was then changed to capecitabine plus irinotecan and, ultimately, capecitabine, irinotecan, plus cetuximab, but his tumor continued to progress through each of these regimens, as determined by an increasing size of multiple hepatic metastases (Table 1). As a final palliative treatment effort, a brief course of regorafenib was initiated, but was poorly tolerated by the patient and quickly discontinued.

**Table 1.**
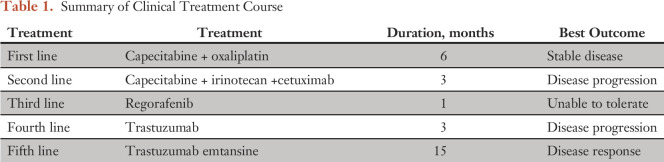
Summary of Clinical Treatment Course

## MATERIALS AND METHODS

Quantitative PCR was performed using the Bio-Rad QX100 droplet digital PCR (ddPCR) system (Bio-Rad, Pleasanton, CA). We used a standard set of HER2-specific Taqman primers and probes (Thermo Fisher Scientific, Foster City, CA) compared with standard references using an ultraconserved region on chromosome 1. Briefly, TaqMan PCR reaction mixtures were assembled using 2X ddPCR Supermix for probes, 20X assays (18 μM primers and 5 μM probe) and restriction digested DNA samples (Bio-Rad). To assess HER2 copy number, 125 ng of each tumor DNA sample was digested with 1.25 units of BsaJI (New England Biolabs, Ipswich, MA) in 15 μL for 1 hour at 60°C. The digests were diluted 1.67-fold to 25 μL with nuclease free water then 25 ng (5 μL) was assayed per 20 μL of ddPCR reaction.

HER2 assay sequences were as follows: forward primer: 5′-LM TO INSERT-3′; reverse primer: 5′-LM TO INSERT-3′; and probe: 5′-FAM-TCTTGGTCGTGTTCTTCATTCGGCACAG-BHQ1-3′. The HER2 assay was duplexed with a standard reference sequence on chromosome 1. This standard reference assay used the following primers: forward primer: 5′-TGAGGGATTCGGCAGATGTTG-3′; reverse primer: 5′-CTGAAAGGCTGGACTTGACAGA-3′; and probe: 5′-VIC-ACTGTGTGCTGGACCT-MGB-3′. All assay primers were ordered from Integrated DNA Technologies (Coraville, IA). Thermal cycling conditions were 95°C for 10 minutes (1 cycle); 94°C for 30 seconds and 60°C for 60 seconds (40 cycles); 98°C for 10 minutes (1 cycle), and a 12°C hold. HER2 copy number per cell was estimated as the ratio of the HER2 and RNA polymerase 30 (*RPP30*) concentrations multiplied by two to account for the two copies of *RPP30* that are expected per diploid genome. Analysis of the ddPCR data was performed using the CNV mode of the QX100 analysis software (version 1.2.9.0; Bio-Rad). Quadruplicate ddPCR wells were analyzed for each sample.

## RESULTS

In the absence of additional treatment options, and given the patient’s good performance status, another liver biopsy was performed and next-generation sequencing was completed in a certified laboratory, which identified several tumor-specific somatic mutations, including APC R232*, *TP53* splice site 97_1G>A, and HER2 amplification. The patient’s case and tumor genomics were then presented to a multi-institutional molecular tumor board for interpretation.

The identification of a mutation in the adenomatous polyposis coli (*APC*) gene was expected and consistent with previous reports in which up to 85% of spontaneous colorectal cancers harbor somatic mutations in the classic tumor suppressor.^16^ A splice site mutation in the *TP53* gene was equally unsurprising, given the relative frequency of *TP53* mutations in human cancers.^17,18^ The molecular tumor board considered neither the *APC* nor *TP53* mutations clinically actionable.

The finding of an HER2 (ERBB2) amplification was unexpected and considered clinically actionable by the molecular tumor board. Cross-reference with The Cancer Genome Atlas confirmed that approximately 4% to 5% of spontaneous colorectal cancers harbor high-level HER2 amplifications,^19^ and preclinical data predicted that anti-HER2 therapy might be clinically effective, particularly when used in a combinatorial approach (Table 2).^20,21^

**Table 2.**
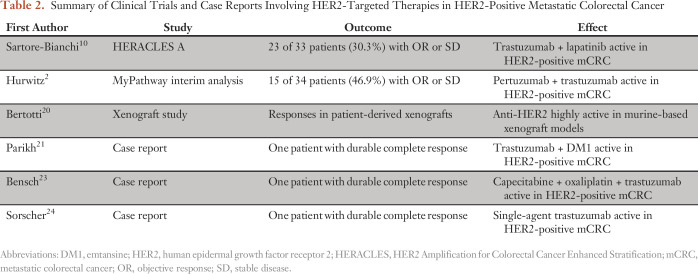
Summary of Clinical Trials and Case Reports Involving HER2-Targeted Therapies in HER2-Positive Metastatic Colorectal Cancer

**Fig 1.**
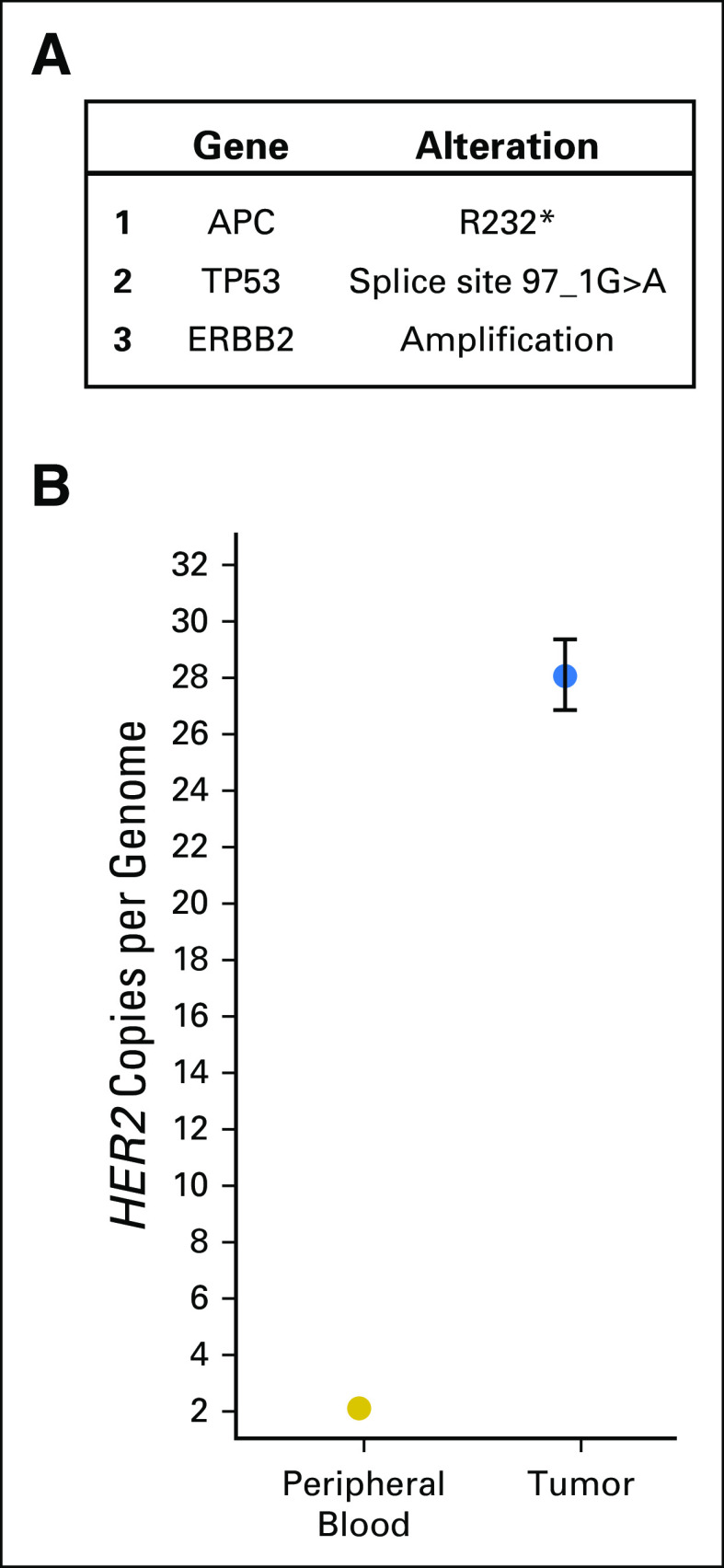
Tumor-specific somatic alterations and quantification of the *HER2* copy number alteration. (A) Next-generation sequencing identified three validated alterations in the patient’s hepatic metastatic colon cancer. (B) Using a droplet digital polymerase chain reaction assay, the total copies of *HER2* per genome in the patient’s metastatic tumor (blue dot) were measured and compared with the total copies of *HER2* in the patient’s germline (peripheral blood, gold dot). Error bars represent standard error of the mean.

To further characterize the HER2 amplification and to validate the finding using an orthogonal method, a ddPCR assay was used. This analysis revealed a HER2 copy number of approximately 30 (Fig 1). In contrast, a control gene, *RPP30*, had the expected diploid copy number of two (Fig 1).

**Fig 2.**
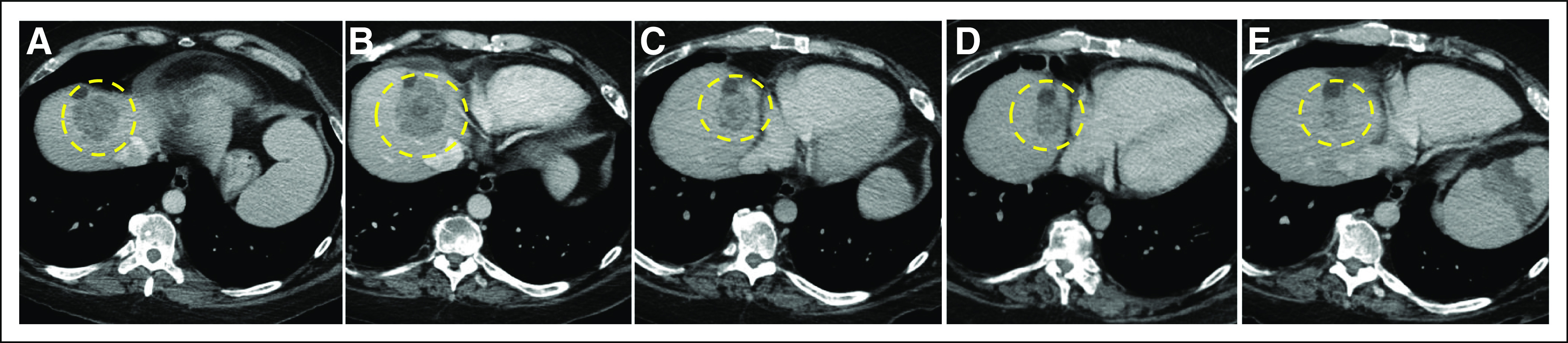
Radiographic evaluation of a hepatic metastasis over the course of treatment. A target metastatic lesion in the patient’s liver was measured by serial computed tomography scans to assess response to treatment. (A) The hepatic target lesion upon conclusion of standard chemotherapy measured 4.8 cm. (B) After 2 months of trastuzumab therapy, the target lesion measured 5.3 cm. (C) After 3 months of treatment with ado-trastuzumab emtansine (T-DM1), the target lesion measured 3.5 cm. (D) After a total of 6 months of treatment with T-DM1, the target lesion measured 3.0 cm. (E) After a total of 12 months of treatment with T-DM1, the target lesion measured 2.2 cm. The dashed gold line indicates the hypodense metastatic target lesion.

Given the genomic findings and the patient’s clinical circumstances, treatment with trastuzumab was initiated. After appropriate initial clinical testing, including echocardiogram, the patient received trastuzumab infusions every 21 days for three total treatments. Follow-up CT imaging, compared with baseline evaluation, revealed disease progression in the liver, with the largest lesion measuring 5.8 cm (Figs 2A and 2B).

Trastuzumab treatments were discontinued and the patient then received trastuzumab-emtansine (T-DM1) infusions every 21 days for three total treatments. Follow-up imaging revealed the largest hepatic metastasis measured 4.6 cm, compared with 5.3 cm at the initiation of T-DM1 (Fig 2C). Three additional infusions at 21-day intervals with subsequent imaging yielded ongoing disease response, with the largest hepatic mass measuring 3.2 cm (Fig 2D). The patient ultimately received T-DM1 infusions every 21 days for 15 months without any discernible negative clinical effect. The largest hepatic metastasis decreased to 2.1 cm (Fig 2E) and two other metastases responded completely. Ultimately, the patient’s hepatic metastatic disease progressed, treatment was discontinued, and the patient died while in hospice care.

## DISCUSSION

This report outlines the impressive clinical course and positive outcome of a patient with metastatic colon cancer treated with a precision oncology approach. The use of next-generation sequencing panels to identify actionable mutations and guide treatment has become increasingly scrutinized as a potentially viable approach for improving outcomes in a variety of cancer subtypes, including lung cancer and melanoma. However, examples of genomics-directed targeted treatment yielding positive responses in refractory colorectal cancer have been rare. Recent clinical data provide encouraging evidence that anti-HER2 therapy can provide clinical benefit in HER2-positive mCRC. Combination therapy, such as trastuzumab plus lapatanib, or T-DM1, as used in this case, appears to be more effective than monotherapy.^10,11,20^ Results recently reported from the HER2 Amplification for Colorectal Cancer Enhanced Stratification (HERACLES A) trial confirm the clinical activity of combination anti-HER2 therapy in mCRC. These findings are bolstered by an interim analysis report from the MyPathWay study^2^ investigating the use of the combination of trastuzumab plus pertuzumab in various solid tumors, including mCRC.

The case reported here suggests that genomics-based analysis of refractory colorectal cancer can lead to effective targeted treatments. The response of this patient with HER2-positive mCRC to anti-HER2 therapy is particularly relevant given recent findings from the HERACLES A trial that 5% of patients with *KRAS* wild-type mCRC harbor a HER2 amplification, as measured by immunohistochemistry,^10^ whereas other studies note a similar prevalence of HER2-positive mCRC (Table 2).^22^ The effect of anti-HER2 therapy in this case is consistent with recent early clinical studies and other case reports of durable responses to HER2-targeted therapy in mCRC (Table 2).^20,21,23,24^

The robust response of this individual’s tumor to genomics-based targeted therapy stands in stark contrast to the disease progression this patient experienced after each of four separate standard cytotoxic chemotherapy regimens. The sustained clinical response documented in this case, and the dramatic clinical improvement, are unusual for patients whose disease has rapidly progressed through previous therapies. The expected progression-free survival and overall survival of a patient with mCRC with refractory disease is approximately 1.7 and 5 months, respectively,^25^ in contrast to the 15-month progression-free survival observed in this case.

Additional data and outcomes are required to confirm that precision oncology routinely yields superior outcomes; however, this report illustrates an additional treatment option in patients with refractory disease who have exhausted all of the commonly used therapeutics for their respective disease and yet retain an acceptable performance status and desire additional treatment—a common clinical scenario. The approach used in this report—genomic analysis in a certified laboratory, molecular interpretation by a board of experts, and subsequent targeted treatment—represents a functional model that has been suggested by others^26^ and can be clinically implemented for the practice of personalized or precision cancer medicine.
